# Associations between physical work environment, workplace support for health, and presenteeism: a COVID-19 context

**DOI:** 10.1007/s00420-022-01877-1

**Published:** 2022-05-16

**Authors:** Emelia Danquah, Nestor Asiamah

**Affiliations:** 1 Department of Procurement and Supply Science, Koforidua Technical University, Koforidua, Eastern Region Ghana; 2 Division of Interdisciplinary Research and Practice, School of Health and Social Care, University of Essex, Wivenhoe Park, Colchester, CO4 3SQ UK; 3 Department of Health Promotion, Africa Centre for Epidemiology, P. O. Box AN 16284, Accra Ghana, Accra North, Ghana

**Keywords:** Physical work environment, Presenteeism, Workplace support for health, Ghana, COVID-19

## Abstract

**Objective:**

Presenteeism has, in a larger sense, been viewed as a negative behaviour, although a limited body of studies suggests and reports its positive implications in an organizational context. This study assessed the association between the physical work environment (PWE) and presenteeism as well as the moderating influence of workplace support for health (WSH) on this relationship.

**Methods:**

This study adopted the cross-sectional design alongside a sensitivity analysis and techniques against common methods bias. The study population was employees of private and public organizations in Accra, Ghana. A total of 590 employees participated in the study and hierarchical linear regression was used to present the results.

**Results:**

PWE had a positive relationship with presenteeism (*β* = 0.15; *t* = 3.04; *p* < 0.05), which means that higher presenteeism was associated with larger PWE scores. WSH positively moderated the relationship between PWE and presenteeism (*β* = 0.23; *t* = 4.84; *p* < 0.001).

**Conclusions:**

Organizations with more satisfactory work environments may serve as preferred protective places for employees during a pandemic, more so within organizations with higher WSH. Interventions rolled out to improve PWE and to provide WSH can attenuate the potential negative influences of presenteeism on individual health and organizational productivity.

## Introduction

Presenteeism refers to a phenomenon of employees who despite being present at work, are unable to be fully engaged in the work environment (Lack 2011). It is the growing propensity for workers to spend more time at work because of insecurity and fear of job loss (Chapman 2005). Some of the key causes of presenteeism are fear of losing a job, high job demands, and low replaceability regarding a job (Aronsson et al. 2000; Biron 2006; Lack 2011). Presenteeism has been seen as a deviant behaviour because it negatively affects employees and organizations (Rainbow et al. 2020; Lack 2011); it can aggravate employee stress, anxiety, and specific health conditions that often require more costly long-term exemptions from work. Since health and wellbeing are major determinants of employee productivity, presenteeism can be a major cause of employee underperformance. Research to date has shown that employee underperformance linked to poor health and wellbeing is the primary consequence of presenteeism faced by organizations (Rainbow et al. 2020; Turpin et al. 2004).

Apart from the above three causative factors of presenteeism, many other factors relating to the individual and job design have been mentioned in the extant literature (Rainbow et al. 2020; Lack 2011). We are concerned that the physical work environment (PWE), hereby defined as attributes such as spatial dimension, architectural design, office ambience, resources, and visual (e.g., aesthetic) factors that exist in and around the workspace (McCoy and Evans 2005), has little recognition in the literature as a potential determinant or, at least, a correlate of presenteeism. To illustrate, employees who, because of ill-health or complaints, should be absent from work, may continue to turn up at work to utilize PWE resources that may not be available at home. While this behaviour may be defiant, it is a well-fated quest for protection or a better life as the individual experiences ill-health or health risks. According to Thayer et al. (2010), organizations with high PWE provide satisfactory work settings characterised by desirable factors such as ventilation, cleanness, space, resources (e.g., equipment, office supplies), and aesthetics. We have observed that employees in developing countries do not have access to these and related workplace resources at home; hence, they may continue to be present at work to utilize or benefit from them when expected to spend some time off their job. If so, presenteeism can be positively associated with PWE. This relationship can be more significant in a COVID-19 context where employees were likely to take sick leave or experience conditions that might warrant their exemption from work.

In response to the outbreak of COVID-19 in Ghana, three cities including Accra were locked down to contain the virus (Asiamah et al. 2021). Research to date (Kumar and Nayer 2021; Cullen et al. 2020; Asiamah et al. 2021) has shown that many employees experienced declines in mental health linked to COVID-19-related psychological and emotional problems. While many employees may have faced situations warranting their exemption from work, other concerns (e.g., domestic violence, not having access to workplace resources at home) may have compelled them to continue turning up at work (Kumar and Nayer 2021; Asiamah et al. 2021). The study of Asiamah et al. (2021) found incidences of domestic violence and related household issues among those observing social distancing in Ghana. Moreover, many employees in developing countries may not have access to PWE resources (e.g., ambience, air-conditioning, car parks, a fast internet) at home, which may result in their presenteeism. Thus, presenteeism during the COVID-19 era can be partly due to factors that may cause employees to resort to their places of work to access resources and escape domestic issues. From this viewpoint, presenteeism is likely to be due to the said attractive PWE factors and could, therefore, have a positive rather than a negative association with PWE in a COVID-19 setting. Given the paucity of studies examining the PWE-presenteeism nexus in a COVID-19 context, this study was conducted.

Further to the above, many organizations provided Workplace Support for Health (WSH) during the COVID-19 pandemic (Awada et al. 2022). WSH is defined as workplace interventions instituted by organizations to inform healthy behaviours and to protect employees against occupational health risks (Kava et al. 2021). WSH includes support for health during the COVID-19 pandemic in the form of social distancing protocols and health education for staff (Awada et al. 2022; Asiamah et al. 2021). In the light of these efforts, WSH may modify the PWE-presenteeism nexus to provide new implications for occupational health and its promotion. This is to say that the negative association between PWE and presenteeism as reported by most previous studies may be different in a COVID-19 context where PWE is operationalized according to Lack’s (2011) definition. If so, there may be several implications for employee health and organizational performance. It is in the interest of these implications that we attempted to examine the following research questions: *(1) is there a significant association between presenteeism and PWE,* and (2) *does WSH moderate the relationship between presenteeism and PWE among employees required to work at home?* We draw on findings from this study to discuss relevant implications for employee wellbeing, organizational performance, and further research.

## Methods

### Design

This study adopted a cross-sectional design including previously used techniques against common methods bias (CMB) and sensitivity analysis.

### Participants and selection

Participants of the study were full-time employees of service and manufacturing companies in Accra, Ghana. Employees of 34 organizations (manufacturing = 14; services = 20) in Accra participated in this study. The selection criteria were: (1) being a full-time employee in a private or public organization; (2) having at least a basic educational qualification (e.g., basic school leaving certificate), which was an indicator of the ability to speak and write English, the medium in which questionnaires were administered; and (3) willingness to participate in the study. A total of 651 employees from private and public sector organizations in Accra volunteered to participate and met the selection criteria. To select participants, we used contact information in a database provided by participating organizations to reach staff. Through this call, we screened for those who met the inclusion criteria and were willing to participate voluntarily. Those who verbally agreed to participate were sent the study’s informed consent statement via email. These individuals completed, signed, and returned the form to formally confirm their involvement in the study. We employed the G*Power 3.9.4 software to calculate the minimum sample size required. We used statistics (i.e., effect size = 0.2, power = 0.8, significance level = 5%, the maximum number of predictors = 9) from a recent study conducted in Ghana (Asiamah et al. 2021) to arrive at the minimum sample size of 88 for the study. To maximize representativeness, data were collected on all 651 employees who met the inclusion criteria.

### Measures and operationalization

This study involved three main variables, namely PWE, presenteeism, and WSH. These constructs were measured by asking participants to respond based on situations in their organizations or experienced by them during the COVID-19 pandemic, ensuring that these constructs were measured in a COVID-19 context. The PWE was measured with a 12-item scale with five descriptive anchors (i.e., 1—strongly disagree, 2—disagree, 3—somewhat agree, 4—agree, 5—strongly agree) that was previously used by Thayer et al. (2010). It produced a satisfactory Cronbach’s alpha ≥ 0.8 in this study. Appendix A shows items of the scale used to measure PWE. Presenteeism was measured with the Stanford 11-item presenteeism scale adopted in whole from Turpin et al. (2004). This tool has two descriptive anchors (i.e., no—0, yes—2) and produced a satisfactory internal consistency (Cronbach’s alpha = 0.97) in the study of Turpin and colleagues. In the current study, it produced a Cronbach's alpha ≥ 0.8. Appendix B shows items of the scale used to measure presenteeism. Workplace support for health was measured with a 5-item scale adopted from Kava et al. (2021) with five descriptive anchors (i.e., 1—strongly disagree, 2—disagree, 3—somewhat agree, 4—agree, 5—strongly agree). This scale produced a satisfactory internal consistency (Cronbach’s alpha = 0.82) in the study of Kava et al. as well as in the current study (Cronbach’s alpha = 0.86). Appendix C shows the items used to measure WSH. Each of the above measures was used because it produced satisfactory psychometric properties. These scales are also relatively short and were therefore easy to complete.

### Identification and measurement of covariates

Potential confounding variables or covariates are variables that can affect the primary predictor in a relationship and can, therefore, affect the primary effect of interest (Asiamah et al. 2019). These variables are also among the major threats to internal validity, especially in cross-sectional designs (Skelly et al. 2012; Asiamah et al. 2019). Related studies (Colenberg et al. 2021; Nasser and Miltagy 2017; Lund et al. 2006; Thayer et al. 2010) have shown that organizational factors such as PWE where people work depend on gender and educational attainment; men and women as well as highly educated and lowly educated people have different opportunities to work in organizations with highly satisfactory PWE. Similarly, whether an individual would work in a highly satisfactory PWE can depend on age, health status, job income, job tenure, physical functional status (PFS), and chronic disease status (CDS) (Cantor 1975; Thayer et al. 2010; Bergefurt et al. 2022). This being so, these personal variables can affect PWE and, therefore, the causal path between it and presenteeism. Hence, we measured these variables as potential covariates. Education was measured as number of years of schooling or formal education. A single item adopted from Asiamah et al. (2021) was used to measure PFS as the extent to which the individual could perform physical tasks unaided. CDS was measured based on Asiamah et al. (2021) by asking participants to indicate the number of clinically diagnosed chronic conditions they had. Remote work time (RWT) was measured by asking employees to report the amount of time (in hours) they spent working from home on a typical weekday.

### Questionnaire structure and CMB attenuation

A self-administered questionnaire comprising four main sections was used to collect data. The first section of the questionnaire presented demographic and potential confounding variables as well as official time spent at home working while the second section presented measures on PWE. The third and fourth sections presented measures on presenteeism and WSH respectively. Preceding these sections was an introductory statement emphasizing the study's ethical requirements and instructions for completing the questionnaire.

In harmony with recommendations in the literature (Jordan and Troth 2019; Pannucci and Wilkins 2010), two steps were taken to avoid or minimize CMB, a primary threat to the internal validity of cross-sectional studies (Jordon and Troth 2019). Firstly, the main sections of the questionnaire were made distinct and independent of the others. This was done by separating sections with preambles to each scale or section that detailed instructions for responding accurately. This first step ensured that participants did not apply perceptions and ratings from the preceding scale to the next scale. The second step is a statistical procedure involving the use of exploratory factor analysis (EFA) with varimax rotation to assess the factor structure of all scales used. This step confirms minimal or no CMB if each scale produces more than one factor in its factor solution (Jordan and Troth 2019). Our EFA shows that all scales produced at least two factors in their factor solutions: PWE—3 factors; WSH—2 factors, and presenteeism—3 factors.

### Data collection approach

This study was approved by an institutional ethics committee (review number provided in the appendix) in Accra, Ghana. Management of the participating organizations also approved the study. All participants consented to participate in the study after reviewing the study’s objectives and ethics statement. Questionnaires were administered through a courier who distributed questionnaires in sealed and stamped envelopes with two research assistants. Participants received and completed questionnaires at home. Before data collection, we contacted the senior administrators of the organizations to agree on our data collection strategy. Participants were required to complete the questionnaires instantly; however, those who were busy and could not complete the survey instantly were given two weeks to return completed questionnaires. Data were collected over 4 weeks (July 12 to August 7, 2021). A total of 611 questionnaires were completed and returned, out of which 590 were analyzed; 21 questionnaires were discarded because they were not completed or were completed halfway.

### Statistical analysis

Data were analyzed using version 28 of SPSS (Statistical Package for the Social Sciences). Data were analysed in two phases, with the first phase focusing on exploratory data analysis whereas the second phase employed hierarchical linear regression (HLR) analysis to address the research questions. The exploratory analysis started with a descriptive analysis in which descriptive statistics were used to summarize the data; frequencies were used to summarize categorical variables whereas the mean (and its standard deviation) was used to summarize continuous variables. We realized based on the summary statistics that four categorical variables were associated with up to 7% missing data. Nevertheless, these missing data were randomly distributed, with less than 1% of them arranged consecutively. Following the work of Asiamah et al. (2021), therefore, we analyzed the data with these missing items. Subsequently, we assessed basic assumptions governing the use of HLR, namely linearity of the primary relationships tested, normality of the data associated with the dependent variable, independence-of-errors, and multi-collinearity. We confirmed the normality of the data with the Shapiro–Wilk’s test at *p* = 0.62. This result confirmed the absence of outliers in the data. To assess the linearity of the primary relationships, we plotted standardized residuals against standardized predicted values of the dependent variable in all models through which the primary relationships were assessed (Garson 2012). These graphs evidenced the linearity of the primary relationships. The independence-of-errors and multicollinearity assumptions were also met through the regression models fitted to assess the primary relationships. The exploratory analysis included a sensitivity analysis aimed at screening the ultimate confounding variables for our regression analyses. Based on a recently used procedure (Asiamah et al. 2021), three of the measured confounding variables (i.e., CDS, age, and RWT) were retained as the ultimate confounding variables.

**Fig. 1:**
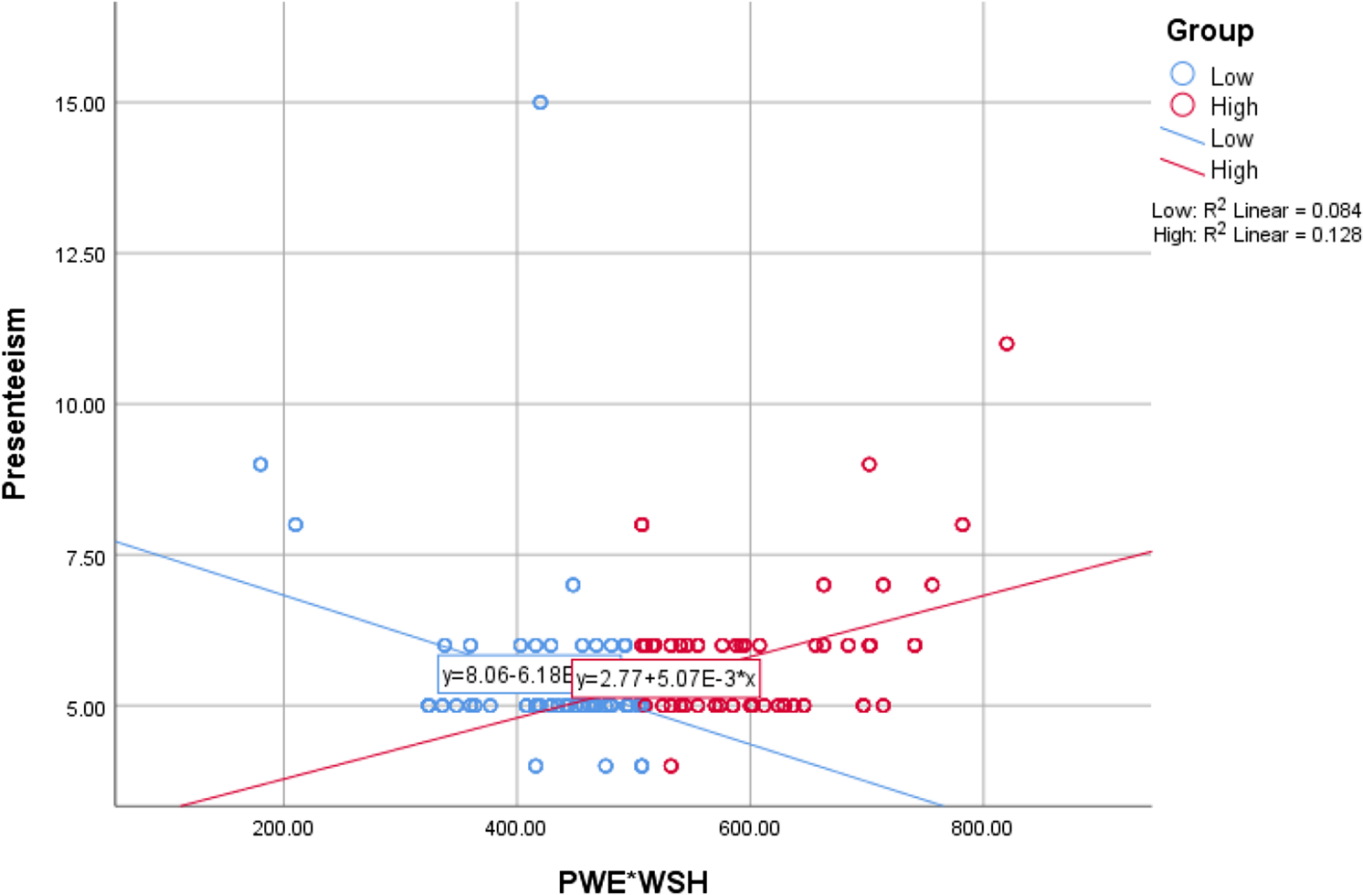
The relationship between presenteeism and different levels (low = 295; high = 295) of the interaction between PWE and WSH. *PWE* physical work environment; *WSH* workplace support for health

In the second phase, the two research questions were addressed with two groups of regression models. The first group comprised two sub-models that examined the association between PWE and presenteeism. The first of these models (i.e., model 1a; first baseline model) did not adjust for the ultimate covariates whereas the second one (i.e., model 1b; first ultimate model) adjusted for the ultimate covariates. The second group (i.e., Models 2a and 2b) assessed the moderating influence of WSH on the association between PWE and presenteeism, whereby models 2a and 2b served as the baseline and ultimate (adjusted) models respectively. The ultimate models served as the source of our final findings. As part of our sensitivity analysis, we compared the baseline and adjusted models to understand the potential effects of the confounding variables on the primary relationships. In testing the moderating role of WSH, we followed Asiamah et al. (2021) to compute a dummy variable representing the interaction between PWE and WSH (i.e., PWE*WSH). A graph (Fig. 1) depicting this interaction was then created after assessing the association between this interaction term and presenteeism. Our analysis focused on *pure moderation*, which means we were interested only in how much the strength of the association between PWE and presenteeism was increased or decreased by WSH. Before fitting the foregoing models, we evaluated the correlation between relevant variables with Pearson’s correlation test. The statistical significance of our result was detected at a minimum of *p* < 0.05.

## Results

Table 1 shows descriptive statistics on the personal characteristics of respondents. In this table, about 48% (*n* = 285) of participants were men whereas about 49% (*n* = 290) were women. In addition, about 64% (*n* = 380) of the participants reported having one or more chronic conditions. The average age of participants was about 35 (Mean = 35.38; SD = 9.68) whereas the average physical function was about 3 (Mean = 2.74; SD = 0.85). Summary statistics on other variables are shown in Table 1. Table 2 shows the results of the sensitivity analysis. In the first stage of the analysis, three variables (i.e., gender, education, and income) were removed from the analysis. In the second stage, only physical function produced less than 10% of the per cent change in beta (β). Thus, CDS, age, and RWT qualified as the ultimate confounding variables.

**Table 1:** Summary statistics of relevant variables

Variable	Category	Frequency/Mean	Percent (%)/SD
Gender	Male	285	48.31
	Female	290	49.15
	Missing	15	2.54
	Total	590	100.00
CDS	None	170	28.81
	≥ 1	380	64.41
	Missing	40	6.78
	Total	590	100.00
Physical functional status	–	2.74	0.85
Education (yrs)	–	18.22	4.21
Income (GhC)	–	1922.44	102.11
Remote work time (hrs)	–	3.21	1.01
Age (yrs)	–	35.38	9.68
Physical Work Environment	–	39.17	4.98
Presenteeism	–	5.64	1.35
WSH	–	14.36	2.28

Mean and SD are for continuous variables whereas frequency and percent are for categorical variables; – Not applicable, *SD* standard deviation, *GhC* Ghana Cedis; *CDS* chronic disease status, *WSH* workplace support for health

**Table 2:** Key findings from the sensitivity analysis

Predictor	Stage 1			Stage 2		
	*β*	*t*	*p*	Adjusted *β*	Change in *β*	% Change in *β*
PWE^a^	0.096	2.35	0.019	–	–	–
Gender (ref. = female)^b^	− 0.01	− 0.26	0.793	–	–	–
Education^b^	− 0.07	− 0.82	0.415	–	–	–
Income^b^	− 0.04	− 0.53	0.598	–	–	–
PFS^c^	0.15	2.82	0.005	0.097	0.001	1%
CDS (reference = ≥ 1)^d^	0.10	1.63	0.105	0.17	0.074	77%
Age (yrs)^d^	− 0.11	− 1.82	0.070	0.134	0.038	40%
RWT^d^	0.14	2.51	0.012	0.124	0.028	29%

*PWE* physical work environment, *CDS* chronic disease status, *PFS* physical functional status, *RWT* remote work time,  – Not applicable

^a^Physical work environment serving as the predictor of presenteeism at stage 1

^b^Variables removed in the first stage of the sensitivity analysis

^c^Variable removed at the second stage of the analysis

^d^Ultimate confounding variables or variables retained for the actual analysis

Table 3 shows the correlation matrix of relevant variables. In this table, there is a positive but weak correlation between PWE and presenteeism (*r* = 0.096; *p* < 0.05; two-tailed), which connotes that larger scores of presenteeism are associated with larger scores of PWE. Chronic disease status (*r* = 0.085; *p* < 0.05; two-tailed) and RWT (*r* = 0.232; *p* = 0.000; two-tailed) are also positively correlated with PWE as ultimate confounding variables. The interaction term between WSH and PWE is also positively correlated with presenteeism (*r* = 0.126; *p* = 0.000; two-tailed).

**Table 3:** Correlation matrix of relevant variables

Variable	#	1	2	3	4	5	6	7
PWE	1	1	.283**	.096*	.731**	.085*	− 0.024	.232**
WSH	2		1	0.077	.856**	− .124**	− .199**	.168**
Presenteeism	3			1	.126**	− .246**	− .179**	− .105*
PWE*WSH	4				1	− 0.059	− .165**	.241**
CDS	5					1	.443**	.128**
Age (yrs)	6						1	.207**
RWT	7							1

*PWE* physical work environment, *WSH* workplace support for health, *RWT* remote work time, *CDS* chronic disease status

^**^p < 0.001; *p < 0.05

Table 4 shows the results from the HLR analysis. In the first baseline model (i.e., model 1a), PWE has a positive association with presenteeism (*β* = 0.1; *t* = 2.35; *p* < 0.05), which confirms the above correlation between presenteeism and PWE. In the first ultimate model on which the conclusions of this study are based (i.e., model 1b), this association is stronger (*β* = 0.15; *t* = 3.05; *p* < 0.05). In the second baseline model (i.e., model 2a), the interaction term (i.e., PWE*WSH) has a positive association with presenteeism (*β* = 0.13; *t* = 3.09; *p* < 0.05). In the second ultimate model (i.e., model 2a), the interaction standardized coefficient increases from 0.13 to 0.23 (*β* = 0.23; *t* = 4.84; *p* = 0.000). A comparison of the two ultimate models suggests that the regression coefficient between PWE and presenteeism (i.e., *β* = 0.15) increases to *β* = 0.23 due to the interaction of WSH with PWE. Thus, WSH improves the strength of the association between PWE and presenteeism by about 53%. Figure 1 depicts the association between the interaction term (i.e., PWE*WSH) and presenteeism.

**Table 4:** The association between physical work environment, workplace support for health, and presenteeism

Model	Predictor	Coefficients			95% CI	Tolerance	Model fit			
		*B*	SE	*β* (*t*)			R^2^	Adjusted R^2^	Durbin-Watson	*F*-test
1a	(Constant)	4.62	0.44	(10.63)**	± 1.71	–	0.009	0.008	–	5.5*
	PWE	0.03	0.01	0.10 (2.35)*	± 0.05	–				
1b	(Constant)	4.97	0.63	(7.88)**	± 2.48	–	0.083	0.074	2.11	9.19**
	PWE	0.05	0.02	0.15 (3.04)*	± 0.06	0.98				
	CDS	− 0.69	0.17	− 0.22 (− 4.02)**	± 0.67	0.76				
	Age (yrs)	− 0.01	0.01	− 0.06(− 1.16)	± 0.03	0.75				
	RWT	− 0.06	0.15	− 0.02 (− 0.38)	± 0.58	0.92				
2a	(Constant)	4.89	0.25	(19.66)**	± 0.98	–	0.016	0.014	–	9.53*
	PWE*WSH	0.00	0.00	0.13 (3.09)*	± 0.00	–				
2b	(Constant)	4.99	0.49	(10.23)**	± 1.92	–	0.114	0.105	2.02	12.98**
	PWE*WSH	0.00	0.00	0.23 (4.84)**	± 0.00	0.95				
	CDS	− 0.59	0.17	− 0.19 (− 3.52)**	± 0.66	0.77				
	Age (yrs)	− 0.01	0.01	− 0.04 (− 0.71)	± 0.03	0.74				
	RWT	− 0.11	0.15	− 0.04 (− 0.75)	± 0.57	0.92				

*SE* standard error (B), *CI* confidence interval, *PWE* physical work environment, *CDS* chronic disease status, *RWT* remote work time, *WSH* workplace support for health

– Not applicable; **p < 0.001; *p < 0.05

## Discussion

This study evaluated the association between PWE and presenteeism in a COVID-19 context as well as the moderating role of WSH in this relationship. Relevant potential covariates were adjusted for in assessing these relationships.

The results of this study indicate that PWE is positively associated with presenteeism, which means that presenteeism was higher among employees reporting larger PWE scores. Similarly, presenteeism was higher in physical workplaces more characterized by aesthetics, walkable spaces, car park, resources for working (e.g., computers, printers) and ambience. This result is, to some extent, analogous to outcomes from several empirical studies (Musich et al. 2006; Merrill et al. 2012; McGregor et al. 2014, 2018) conducted around the world. McGregor and colleagues (2014), for instance, found a positive association between work environment and presenteeism with data from Europe and North America. Merrill et al. (2012) have also reported a positive association between work environment and presenteeism in the US. Musich et al. (2006) and McGregor et al. (2018) reported related findings with data from multiple countries. These and similar previous studies, nevertheless, related presenteeism to 'work environment' rather than 'physical work environment'. While the 'work environment' is a construct of unfavourable psychosocial factors (e.g., poor organization support, low replaceability, low job security), PWE embodies built environment factors (e.g., aesthetics, ventilation, office beauty, air conditioning, ambience) that make the workplace attractive and satisfactory. So, presenteeism in the context of these previous studies would be owing to unfavourable psychosocial factors such as employees’ fear of losing a job and the inability of the organization to fill the roles of exempt employees (i.e., employees who have been formally exempted from work due to ill-health or related factors). On the flip side, the positive relationship between PWE and presenteeism implies that employees were attracted to their workplaces where essential resources could be accessed. This deduction from our result backs Cantor’s (1975) bio-ecological framework that argues that human ecosystems providing access to aesthetic attributes, services, and other resources encourage social engagement. This reasoning implies that exempt employees are more likely to engage with work if their workplaces are characterized by attractive physical features.

Though presenteeism is considered a negative behaviour (Merrill et al. 2012; McGregor et al. 2018), its positive relationship with PWE can imply opportunities or challenges for an organization. The opportunities are increased staff engagement and additional work output from employees required to be absent from work. These opportunities are consistent with the concept of *therapeutic presenteeism* (Karanika-Murray and Biron 2020), which emphasizes the possibility of employees resorting to the workplace perceived to serve as a protective environment where pro-health resources can be accessed. A recent study carried out by Lohaus et al. (2021) reported several benefits of therapeutic presenteeism to the individual. One of the most pronounced benefits was reported by individuals as “do not want to let the sickness get me down”, which means that this type of presenteeism is a way to recover from a sickness. Lohaus and colleagues also reveal that therapeutic presenteeism enables employees to demonstrate their physical capacity to work while facing ill-health. Finally, therapeutic presenteeism gives employees the opportunity to do something (e.g., buy groceries) on their way to or from work (Lohaus et al. 2021). On the flip side, the organization may face some challenges due to therapeutic presenteeism. These challenges can include overuse of organizational resources and potential role conflicts between exempt employees and those replacing them. If exempt employees are not replaced, their presenteeism may benefit the organization as they may be re-filling the vacant roles they left behind following their formal exemption from work. If, on the other hand, exempt employees use organizational resources to meet personal needs, the organization can lose resources through presenteeism. The above undesirable outcomes fit into a framework of low productivity indicators or outcomes that form a part of the concept of *therapeutic presenteeism*.

Organizations may, therefore, find it helpful to monitor exempt employees returning to work at a time they are mandated to be absent from work. Another potential challenge is low productivity and absenteeism in the long term that are the result of presenteeism. There is a consensus among researchers (Musich et al. 2006; McGregor et al. 2018; Turpin et al. 2020) that employees who continue to work following their official exemption from work owing to ill-health risk more serious health problems that can lead to low productivity or longer sick leaves. So, while organizations may benefit from providing satisfactory physical workplaces that encourage employee engagement, they need to monitor and control how exempt employees use the PWE and its resources, especially during a pandemic. Furthermore, Lack’s (2011) definition of presenteeism, which we adopted in this study, assumes employees’ physical presence at work, but social distancing measures necessitated by the COVID-19 pandemic suggest that employees can be present at work virtually (through working online) or remotely (through working from home). Suffice it to say that presenteeism would not necessarily be about being physically present at work. Since many organizations may maintain hybrid work (i.e., working from home and at the physical workplace), the definition of Lack may evolve in response to global trends accompanied by the pandemic. If so, organizations could find ways to maximize opportunities for health and productivity for hybrid work.

Further to the above, this study found that WSH positively moderated the relationship between PWE and presenteeism, which implies that presenteeism was more positively affected by PWE in organizations where WSH was higher. This outcome of the study is congruent with the salutogenic model (also known as salutogenesis) first coined by Antonovsky (1996). This theory implies that WSH is a form of intervention that would enable employees to remain healthy or avoid health risks. Recent adaptations of this theory in occupational medicine (Bauer 2022) suggest that workplace design that emphasizes improvement in aesthetics, walkability, office ambience, and availability of physical resources is typical of health promotion within organizations that would encourage engagement and increase satisfaction as well as productivity. The foregoing moderating role, thus, signifies the role of WSH in creating conducive and attractive workplaces and endorses the salutogenic model’s explanation of the potential positive relationship between workplace health promotion and employee engagement, which is an implication of presenteeism in the current study context. Furthermore, the moderating role of WSH confirmed in this study affirms workplace health promotion efforts reported recently by Hunter et al. (2021) as intrinsic motivation factors that may fit in the two-factor theory of Herzberg (1964). This reasoning is premised around the fact that the physical environment is an intrinsic factor that can contribute to work satisfaction, depending on whether it offers what employees expect. If the physical workplace features attributes such as aesthetics and essential resources, it can be expected to contribute to work satisfaction, which is an indicator of exempt employees’ availability at work.

Our sensitivity analysis shows that the relationships between PWE, WSH, and presenteeism are affected by personal variables, particularly the ultimate confounding variables retained in the sensitivity analysis (i.e., age, CDS, RWT). This is rightly so because these covariates affected (i.e., decrease or increase) the primary regression weights between the baseline and ultimate models. Our result has two main implications. Firstly, the PWE can be affected by personal demographic variables, which means that future researchers must endeavour to adjust for these and related potential covariates; otherwise, their estimates may be misleading. If we did not control for the ultimate confounders in this study, we would have made conclusions in this study based on estimates from the baseline models.

To add, whether an employee works in an organization with a satisfactory PWE depends on factors such as age and CDS or health status. Thus, companies at a certain level of PWE may prefer employees within certain age groups or with certain health statuses, or employees with some health conditions may prefer organizations with certain PWE conditions. If so, PWE may affect organizational choices as far as personnel are concerned and could be a factor employees consider in selecting their ideal places to work. Supporting this thought are studies (Lund et al. 2006; Thayer et al. 2010; Al Zamel et al. 2020) that have revealed that employees prefer organizations with aesthetic and luxurious settings where resources for health and high performance are readily available. Lund and colleagues added that organizations with beautiful workplaces are more likely to employ highly educated and energetic (young and healthy) employees.

This study has some limitations that we would want to acknowledge to guide future research and decision making. As a cross-sectional design, this study does not establish cause and effect between the variables. Even so, our findings provide associations or regression weights that can be used to calculate sample sizes and power in related future experimental studies, which are the ideal designs for establishing cause and effect between the variables (Asiamah et al. 2019). Though the cross-sectional design is unable to establish cause and effect between variables, it is a reliable source of evidence if it eliminates or minimizes key forms of bias (Jordan and Troth 2019; Asiamah et al. 2019). Interestingly, our effort to control for confounding variables and reduce or eliminate CMB in harmony with STROBE (Strengthening the Reporting of Observational Studies in Epidemiology) guidelines makes this study a useful source of evidence. Even so, we could not have identified and adjusted for confounding variables (e.g., type of job) suited for all contexts. We would also want to admit that, by focusing on a sample of employees in Accra and Ghana, our findings may not represent situations in other settings, especially non-Ghanaian settings. Yet, our calculation of a minimum sample size required for the study may have compensated for this shortcoming. We also admit that our measure of chronic disease status (i.e., the number of chronic conditions the individual had) may be insufficient or incomplete, so future studies may have to use more elaborate measures. In any case, future studies are encouraged to use a larger and more diverse sample that can warrant absolute generalizability. Future replications of this study that yield evidence comparable to our results are encouraged.

## Conclusion

Presenteeism is higher among employees who reported larger PWE scores, regardless of age, CDS, and RWT. This study, therefore, concludes that workplaces with more satisfactory physical work environments are perceived to be associated with higher presenteeism. More interestingly, WSH strengthens the foregoing association between PWE and presenteeism by about 53%. This is to say that PWE more strongly predicts presenteeism in workplaces reporting higher WSH. It can, therefore, be concluded that WSH favours the PWE or contributes to more satisfactory work environments, leading to higher presenteeism. This outcome reveals the possibility of presenteeism increasing as the PWE improves in light of workplace health promotion interventions during the COVID-19 pandemic. So, the adoption of WSH or improved implementation of this programme can make workplaces more attractive and satisfactory, resulting in presenteeism, which can be an indicator of sustained staff engagement in the current context.

## Data Availability

The data used for this paper will be made available by the corresponding author upon reasonable request.

## Code availability

Codes will be made available upon request.
